# Change in the Location of a Pseudotumor Around the C2 Odontoid Process from Posterior to Anterior to the Odontoid Process in the Natural Course: A Case with “Antero-Odontoid Pseudotumor” or “Peri-Odontoid Pseudotumor”

**DOI:** 10.3390/jcm14124182

**Published:** 2025-06-12

**Authors:** Hiroki Takeda, Takaya Imai, Yuki Akaike, Soya Kawabata, Nobuyuki Fujita, Shinjiro Kaneko

**Affiliations:** 1 Department of Spine and Spinal Cord Surgery, School of Medicine, Fujita Health University, Toyoake 470-1192, Aichi, Japan; 2 Department of Orthopedic Surgery, School of Medicine, Fujita Health University, Toyoake 470-1192, Aichi, Japan

**Keywords:** retro-odontoid pseudotumor, antero-odontoid pseudotumor, rheumatoid arthritis, transverse ligament, apical ligaments, alar ligaments

## Abstract

**Background:** A pseudotumor adjacent to the odontoid has been reported to be a non-neoplastic mass that is mainly associated with atlantoaxial instability. **Methods:** Case report. **Results:** A 72-year-old woman presented to our clinic with a chief complaint of bilateral fine motor dysfunction and gait disturbance. She had rheumatoid arthritis as a comorbidity. Physical examination revealed bilateral hand fine motor dysfunction and signs of myelopathy, including hyperreflexia of the deep tendon reflexes in the lower extremities. Magnetic resonance imaging (MRI) showed a retro-odontoid pseudotumor. Surgery was proposed to the patient, but she did not wish to undergo surgery at this time. At a follow-up visit approximately one year after the initial visit, she complained of the progression of the bilateral hand fine motor dysfunction and gait disturbance. MRI demonstrated a pseudotumor in the space anterior to the odontoid process, indicating that the localization of the pseudotumor around the odontoid process changed from the posterior space to the anterior space in its natural course. **Conclusions:** The speculated sequential mechanism of the change in the location of the pseudotumor from the posterior space to anterior space to the odontoid process in the natural course is as follows: As the rheumatoid arthritis progressed, the C1-2 joint was immobilized in the dislocated position, and as a result, the retro-odontoid pseudotumor disappeared due to immobilization of the C1-2 joint. Following the disappearance of the retro-odontoid pseudotumor, the odontoid process shifted backward owing to rupture of the transverse annular ligament. Consequently, a new space appeared in front of the odontoid process. Subsequently, damage to the apical and alar ligaments resulted in pseudotumor formation in the new space. Considering our case, the formation of an antero-odontoid pseudotumor occurs only in limited cases, with extreme progression of the pathology. Most cases of retro-odontoid pseudotumors are treated by surgery before such a progression; therefore, we consider that such a case has not yet been reported in the literature.

## 1. Introduction

With the development of magnetic resonance imaging (MRI), it has become possible to elucidate various pathologies of upper cervical spine diseases. In 1986, Sze et al. first reported three cases with space-occupying masses that had developed behind the odontoid process with subluxation of the annular axis vertebrae and named the space-occupying mass a “pseudotumor” [1]. Since this report, the term “pseudotumor” has become widespread [2,3,4,5,6,7,8]. Retro-odontoid pseudotumors have various clinical manifestations and signs and may lead to progressive myelopathy or sudden death.

A pseudotumor adjacent to the odontoid has been reported to be a non-neoplastic mass that is mainly associated with rheumatoid arthritis (RA) and atlantoaxial subluxation [1,2,3,4,5,6,7,8]. The main etiology of retro-odontoid pseudotumors is the degeneration of the transverse ligament due to chronic mechanical stress [9]. In patients with RA, a commonly reported cause of retro-odontoid pseudotumors is pannus, an inflamed granulation tissue arising from the synovial membrane around the odontoid process. Pannus formation has been uniformly attributed to inflammation of the synovial membrane, which results in the overgrowth of hyaline cartilage and periarticular inflammation. Inflammation, ligamentous laxity, and bone erosion ultimately result in joint subluxation and instability [1,6,10,11]. There have been many reports regarding the reduction or disappearance of retro-odontoid pseudotumors after occipitocervical fusion [4,12]. These reports support the hypothesis that retro-odontoid pseudotumors could result from instability at the C1-2 or craniovertebral junction [3,13,14].

Here, we report a case in which the location of a pseudotumor around the odontoid process changed from the posterior space to the anterior space to the odontoid process during its natural course. To the best of our knowledge, there have been no reports regarding such a case in the previous literature.

## 2. Case Presentation

**Figure 1:**
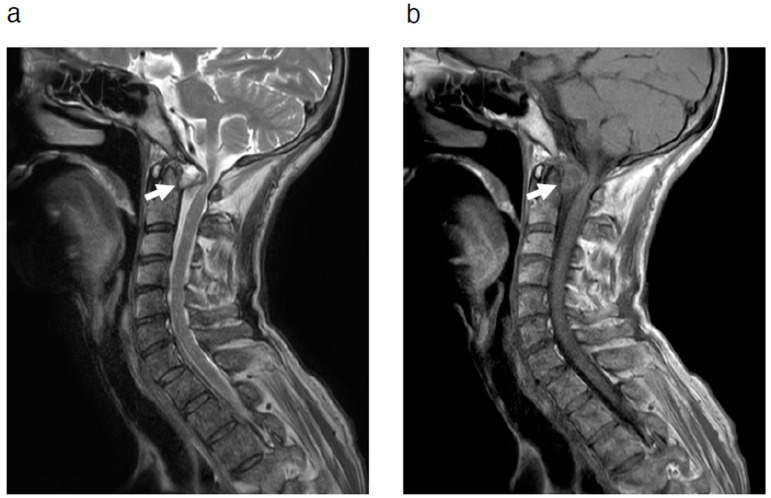
MRI at the initial visit revealed a retro-odontoid pseudotumor (white arrows). Sagittal view of T2-weighted (**a**) and T1-weighted (**b**) images. MRI = magnetic resonance imaging.

A 72-year-old woman presented to our clinic with a chief complaint of bilateral hand fine motor dysfunction and gait disturbance. She had RA as a comorbidity and had been treated with steroid medication for approximately 10 years. Physical examination revealed bilateral hand fine motor dysfunction and signs of myelopathy, including hyperreflexia of the deep tendon reflexes in the lower extremities. MRI demonstrated a retro-odontoid pseudotumor with a relatively large size (Figure 1). Surgery, the standard treatment for a retro-odontoid pseudotumor of this size, was proposed to the patient. Posterior occipitocervical fusion was considered the appropriate surgical procedure, but she did not wish to undergo surgery at that time.

**Figure 2:**
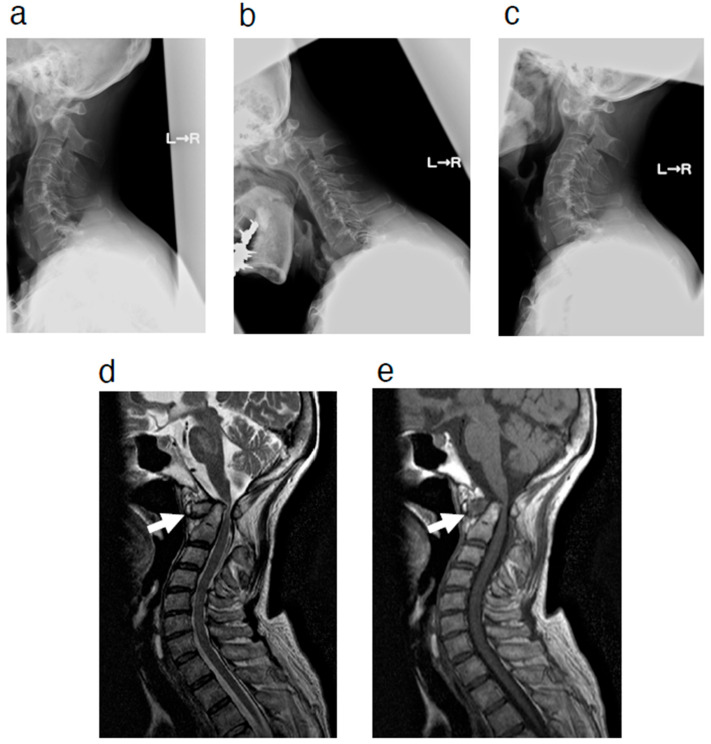
(**a**–**c**) Radiographic image at the follow-up visit. Lateral view in flexion (**a**), neutral (**b**), and extension (**c**) positions. (**d**,**e**) MRI at the follow-up visit showing an antero-odontoid pseudotumor (white arrows). Sagittal view of T2-weighted (**d**) and T1-weighted (**e**) images. MRI = magnetic resonance imaging.

**Figure 3:**
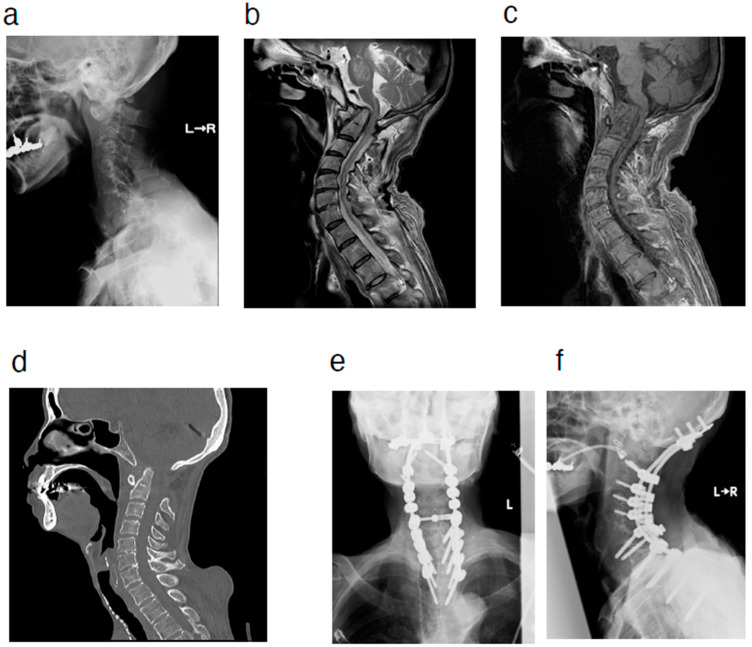
(**a**) Lateral view of radiographic image at readmission. (**b**,**c**) MRI at readmission. Sagittal view of the T2-weighted (**b**) and T1-weighted (**c**) images. (**d**) CT imaging at readmission. Sagittal view. (**e**,**f**) Radiographic images after reoperation. A-P view (**e**) and lateral view (**f**). MRI = magnetic resonance imaging. CT = computed tomography.

At a follow-up visit approximately one year after the initial visit, she complained of the progression of bilateral hand fine motor dysfunction and gait disturbance. Physical examination revealed progression of myelopathy, including bilateral muscle weakness in the upper and lower limbs (3 to 4 by manual muscle testing). On plain radiography, the atlantodental interval was enlarged, which was not observed at the time of the initial visit. However, no obvious instability of the atlantoaxial joint was noted. MRI showed a pseudotumor in the space anterior to the odontoid process, indicating that the localization of the pseudotumor around the odontoid process changed from the posterior space to the anterior space to the odontoid process (Figure 2). This patient had a history of surgery for valvular heart disease and stomach cancer, as well as other comorbidities such as malignant lymphoma and severe emaciation, so the rheumatologist treating this patient for comorbid RA recommended a less invasive procedure. Furthermore, the patient did not want to undergo surgery using implants at this point. Considering various factors, including the patient’s comorbidities, decompression surgery was performed by resecting the posterior arch of the atlas. After the surgery, the patient showed improvement in her preoperative bilateral hand fine motor dysfunction and gait disturbance. Approximately one year after the initial surgery, respiratory dysfunction was observed, and the patient was readmitted to our hospital. Vertical dislocation of the atlantoaxial joint, which was not seen before the initial surgery, was observed. The patient subsequently underwent posterior occipito-cervico-thoracic fusion (O-Th4) surgery (Figure 3). The patient’s symptoms, including respiratory dysfunction, improved after the surgery. After surgery, the patient developed temporary pneumonia, but it improved soon after and she underwent continued rehabilitation at our hospital. Approximately a month later, she was transferred to a hospital closer to her home to continue her rehabilitation; her preoperative symptoms, including respiratory dysfunction, reportedly improved at the new hospital. After that, she moved into a geriatric healthcare facility and did not visit our hospital again.

## 3. Discussion

As described, a pseudotumor adjacent to the odontoid has been reported to be a non-neoplastic mass that is mainly associated with RA and atlantoaxial subluxation [1,2,3,4,5,6,7,8]. Crockard et al. reported five cases of retro-odontoid pseudotumors in the absence of axial subluxation in elderly patients and stated that posterior pseudotumors of the odontoid process were most likely the result of reactive mass formation due to repeated injury to and repair of the transverse ligament, including those caused by axial subluxation [9]. Later, Chikuda et al. reported a radiological analysis of posterior pseudotumors of the odontoid process [6]. In their paper, they proposed a possible etiology of the posterior pseudotumor of the odontoid process without subluxation of the C1-2 joint. According to the paper, a reactive mass might have been formed due to damage to the transverse ligament from excessive mechanical load on the C1-2 joint, accompanied by decreased mobility of the mid and lower cervical spine due to ossification of the anterior longitudinal ligament or other factors such as disk degeneration.

The etiology of retro-odontoid pseudotumors is related to the process of ligament tear around the odontoid [9]. The transverse ligament is the main ligament involved in the stability of the atlantoaxial vertebrae. The reason pseudotumors around the odontoid process are commonly located behind the odontoid process is due to the fact that this is where the transverse ligament is located. There are many ligaments surrounding the odontoid process, and the transverse ligament and its superior and inferior limbs are collectively called the cruciate ligaments. In atlantoaxial subluxation, joint instability results in partial tears of the transverse ligament and reactive changes to repair the injury [2,4,12]. These reactive changes include hypervascularization and formation of fibrocartilaginous tissue [9]. This process leads to degeneration and abnormal thickening of the transverse ligament with mass formation [13]. Besides instability, osteoarthritis of C1-2 itself causes a partial tear or degradation of the transverse ligament, which induces the formation of a retro-odontoid pseudotumor [10].

**Figure 4:**
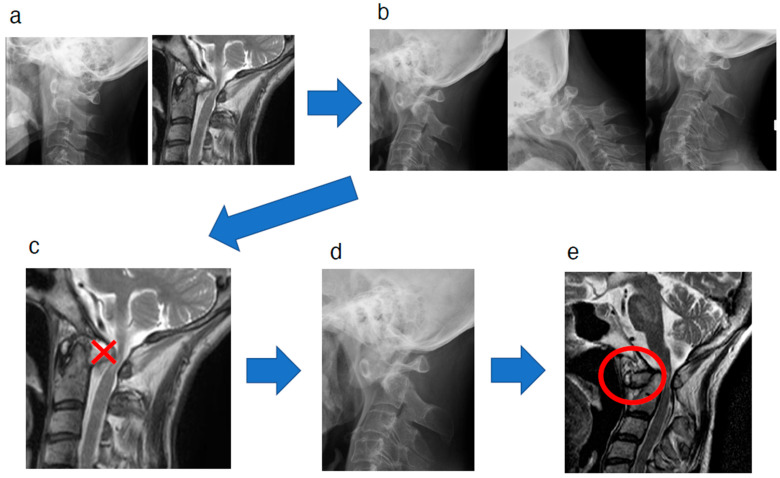
The speculated sequential mechanism of the change in the location of the pseudotumor around the C2 odontoid process from posterior to anterior to the odontoid process in the natural course. The injured transverse ligament was not repaired, resulting in a rupture. As the disease progressed in rheumatoid arthritis, the C1-2 joint was immobilized in the dislocated position (**a**→**b**), and as a result, the retro-odontoid pseudotumor disappeared due to the immobilization of the C1-2 joint (**c**). Following the disappearance of the retro-odontoid pseudotumor, the odontoid process shifted backward and dislocated due to the rupture of the transverse annular ligament. As a result, a new space appeared in front of the odontoid process (**d**). Subsequently, damage to the apical and alar ligaments resulted in pseudotumor formation in the new anterior space (circled in red) (**e**).

As described, we experienced a case in which a pseudotumor was formed anterior to the odontoid process. In this case, we observed that the location of the pseudotumor around the odontoid process changed from the posterior space to the anterior space to the odontoid process. The transverse annular ligament is the most essential ligament to prevent anterior dislocation of the C1-2 joint; therefore, it contributes the most to the stability of the annulus. The speculated sequential mechanism of the change in the location of the pseudotumor around the C2 odontoid process from posterior to anterior to the odontoid process in this case is as follows (Figure 4): In our case, the injured transverse ligament was not repaired, resulting in rupture. As the disease progressed in RA, the C1-2 joint was immobilized at the dislocated position (Figure 4b), and as a result, the retro-odontoid pseudotumor disappeared due to the subsequent immobilization of the C1-2 joint (Figure 4c) [3,12]. Following the disappearance of the retro-odontoid pseudotumor, the odontoid process shifted backward and dislocated because of the rupture of the transverse annular ligament. Consequently, a new space appeared in front of the odontoid process (Figure 4d). Subsequently, damage to the apical and alar ligaments resulted in pseudotumor formation in the new anterior space (Figure 4e).

Here, we also describe our speculation regarding the reason that there have been no reports of “antero-odontoid pseudotumors”. Considering our case, the formation of antero-odontoid pseudotumor occurs only in limited cases with extreme progression of the pathology. Most cases with retro-odontoid pseudotumors are treated surgically before such a progression; thus, we consider that such cases have not yet been reported in the literature. As described, the transverse ligament is the main ligament involved in the stability of the atlantoaxial vertebrae, so the pseudotumor first appears behind the odontoid process following sequential damage to and repair of the transverse ligament [1,9]. Therefore, it is considered that the initial pathology of the antero-odontoid pseudotumor tends to be a retro-odontoid pseudotumor, and antero-odontoid pseudotumors can be observed only at the extremely progressed stage in cases such as ours.

As mentioned above, approximately one year after the initial decompressive surgery, vertical dislocation of the atlantoaxial joint was observed, which was not seen before the initial surgery. The patient then underwent posterior occipito-cervico-thoracic fusion (O-Th4). Although the patient’s symptoms improved after both surgeries, additional surgery may not have been necessary if the initial surgery had been occipitocervical fusion rather than decompression. Based on the sequential findings of this case, we consider that antero-odontoid pseudotumor is only seen at a very advanced stage, and occipitocervical fusion is recommended as a reasonable surgical procedure.

Taken together, spine surgeons should consider that the localization of a pseudotumor around the odontoid process may change over time in some cases and take this into account in the decision-making process for surgical procedures. Additionally, the phenomenon might be better termed as “a peri-odontoid pseudotumor” considering the etiology.

## 4. Conclusions

We experienced a case in which the location of the pseudotumor around the odontoid process changed from the posterior space to the anterior space to the odontoid process in the natural course and described the speculated sequential mechanism of the change. The formation of an antero-odontoid pseudotumor occurs only in limited cases, with extreme progression of the pathology. Most cases of retro-odontoid pseudotumors are treated by surgery before such a progression; therefore, we consider that such a case has not yet been reported in the literature.

Taken together, the localization of a pseudotumor around the odontoid process may change over time in some cases, and spine surgeons should consider this phenomenon. Additionally, the phenomenon might be better termed as “a peri-odontoid pseudotumor” considering the etiology. Further research into this phenomenon is needed in the future to better understand it.

## Data Availability

Data are contained within the article.
